# Mourning and psychotic disorders: A different way to experience the loss

**DOI:** 10.1192/j.eurpsy.2022.907

**Published:** 2022-09-01

**Authors:** L.T. Rodríguez Hernández, M.L. Costa

**Affiliations:** 1 Hospital Universitario Jose Gemain, Psychology, Leganés, Spain; 2 Hospital Universitario Severo Ochoa, Psychiatry, Leganes, Spain

**Keywords:** Classification of mental disorders, Psychopathology, mourning, schizophrénia

## Abstract

**Introduction:**

We present the case of a 48-year-old female patient diagnosed with schizoaffective disorder whose father passed away recently. The patient was facing an appalling mourning which was expressed in the form of behavior disorder and positive psychotic symptoms. Mourning is a natural reaction to the loss of a loved one which involves an internal world transformation, affecting both images of the self and the perceived environment.

**Objectives:**

To analyse the guidelines for mourning approach in chronic psychotic patients.

**Methods:**

A case report is presented alongside a review of the relevant literature regarding mourning in patients with chronic psychotic conditions.

**Results:**

Accepting the loss, working through disruptive emotions, adjusting to a world without the deceased and finding an enduring connection with the loved one are the four tasks of mourning described by Worden. In our case, the patient was immersed in the first two tasks. Difficulties in accepting the loss, tolerating harmful emotions and establishing new affective links were observed, as well as massive projection of unbearable emotions such as sadness, anger, fear and guilt. The available literature identifies these idiosyncrasies as common in the grief processing in patients with chronic psychotic disorders.

**Conclusions:**

In patients with psychosis, difficulties in symbolization, emotional processing and social bonding could have repercussions in the development of grief. However, these features do not imply a pathologic mourning. Tolerating mourning as a normal reaction in psychotic patients is needed, even if the patient expresses non-typical symptoms such as acute psychosis symptoms, hallucinations or behavior disorder.

**Disclosure:**

No significant relationships.

